# Editorial: Perspectives on PTSD and its treatment

**DOI:** 10.3389/fnbeh.2023.1327251

**Published:** 2023-11-06

**Authors:** Jacklynn M. Fitzgerald, Ashley A. Huggins

**Affiliations:** ^1^Department of Psychology, Marquette University, Milwaukee, WI, United States; ^2^Department of Psychology, University of Arizona, Tucson, AZ, United States

**Keywords:** posttraumatic stress disorder (PTSD), trauma, neural, neurobiology, risk

The field of trauma research is rapidly evolving. While historically it has emphasized understanding the development of posttraumatic stress disorder (PTSD) as a single construct, it is now increasingly recognized that the manifestation of PTSD is complex—the disorder is heterogenous in symptom presentation, longitudinal prognosis (e.g., issues of chronicity), and in terms of understanding who is most at risk. More than ever, we readily recognize that there are important mediating and moderating factors that influence how PTSD manifests, including how it operates at the neural level. Further, the manifestation of PTSD may be influenced by many neural disturbances. These points are emphasized by the articles published in this Research Topic.

For example, physical health may be an important factor that impacts mental health outcomes after trauma. In the current issue, Lin et al. explored links between PTSD and asthma by probing the anterior insula, a region key to anxiety sensitivity and interoceptive processing (Muftuler et al., 2011). In a large sample of 508 trauma-exposed women, symptoms of PTSD and depression were significantly higher in those who had a diagnosis of asthma. In a subsample with neuroimaging data, anterior insula response to threat was associated with symptoms of depression, but not for women who had comorbid asthma. Emerging work continues to implicate complex relationships between traumatic stress, the brain, and physical health, particularly conditions related to inflammation (such as asthma; Allgire et al., 2021) and cardiovascular function (Seligowski et al., 2022). Indeed, asthma typically develops during childhood and physical health experiences stemming from early life may predispose trauma survivors for more adverse mental health outcomes in adulthood.

Findings from Huffman et al. published here underscore this point: precisely that adverse childhood experiences (and particularly, the timing of these experiences) impacts PTSD outcomes. In a sample of adult trauma survivors, earlier exposure to adversity was associated with smaller thalamic volumes, which in turn predicted risk for PTSD symptoms in the aftermath of novel trauma. The thalamus, a key sensory hub, appears to have a longer period of development relative to other regions of the brain (Muftuler et al., 2011); earlier adversity may thus be particularly consequential, as environmental exposures that impact thalamic development could have persistent downstream consequences for processing stress that increases risk for PTSD after trauma. Notably, the thalamus is a brain region not readily identified within the “canonical PTSD neurobiological model” which heavily emphasizes disruption in cortico-limbic circuitry and exaggerated limbic region responsivity (Fitzgerald et al., 2018). Principal among these disruptions is amygdala hyperreactivity to internal and external cues that resemble the traumatic event and subsequently trigger fear. Thus, in addition to highlighting the role of early adversity, Huffman et al. demonstrate that abnormalities outside of the canonical neural model governing fear processing are informative for understanding PTSD development.

To this point, Ely et al. here demonstrate that some individuals with PTSD exhibit deficits in attentional processing outside of the domain of emotion. In a mini review, these authors showcase findings across several studies to-date that report on problems in attentional engagement among those with PTSD. Further, these authors found that these deficits were represented at the neural level by increased ventral attention network (VAN) processing. The VAN facilitates bottom-up processing of attention (as opposed to top-down directed attention) and involves circuitry in temporal and parietal cortices as well as inferior/frontal gyri. Findings across studies suggest that attentional problems occur when patients are instructed to attend to predefined salient stimuli and that patients with PTSD were deficient when directed to shift attention. Combined, this suggests that PTSD is influenced by difficulty in disengaging from stimuli once fixated and that this deficit also occurs in response to stimuli that are not emotional (e.g., not threat driven).

Given heterogeneity in the way in which PTSD manifests and is associated with neural aberrations, additional noteworthy neurobiological research on PTSD centers on identifying precisely which circumstances and factors influence treatment success. Given the uniqueness of the disorder, no one treatment may work for all cases. To this point, Hinojosa et al. demonstrate in this issue that treatment success following prolonged exposure (PE) therapy is predicted by baseline neural responding in the amygdala and the ventromedial prefrontal cortex as well as habituation of the amygdala across repeated exposure to emotional face stimuli. Here, these authors found that determining who most benefitted from PE was determined by a neural profile defined by less amygdala response to emotional faces alongside greater vMPFC response and a greater decline in the amygdala over continuous presentation of emotional faces. This suggests that both pre-existing individual differences in neural responsivity as well as differences in how neural responses changed over time were informative. In particular, such findings may help treatment providers consider ways to better model the mechanisms of therapeutic change in the lab, in so much as capturing corollaries of treatment change that occurs over sessions may closely align with time-dependent changes in the brain.

Combined, the articles that appear in this Research Topic further our neurobiological understanding of PTSD and its treatment. First, they expand our understanding of the moderating and pre-existing factors that influence neural aberrations of the disorder, zeroing in on the importance of studying early life experiences and physical health comorbidities. Second, they emphasize that the neurobiological model of PTSD is one whereby both alterations in affective and cognitive processes exist and demonstrate that broad attentional deficits beyond threat processing exist in the disorder. Finally, findings demonstrate that fully predicting treatment success for patients with PTSD may depend on understanding individual differences in brain functioning, precisely in how the brain responds dynamically to stimuli over time.

## Author contributions

JF: Writing—original draft, Writing—review & editing. AH: Writing—original draft, Writing—review & editing.
